# Evidence for Contribution of CD4+CD25+ Regulatory T Cells in Maintaining Immune Tolerance to Human Factor IX following Perinatal Adenovirus Vector Delivery

**DOI:** 10.1155/2015/397879

**Published:** 2015-01-31

**Authors:** Megha S. Nivsarkar, Suzanne M. K. Buckley, Alan L. Parker, Dany Perocheau, Tristan R. McKay, Ahad A. Rahim, Steven J. Howe, Simon N. Waddington

**Affiliations:** ^1^Gene Transfer Technology Group, University College London, 86-96 Chenies Mews, London WC1E 6HXZ, UK; ^2^Institute of Cancer & Genetics, Cardiff University School of Medicine, Heath Park, Cardiff CF14 4XN, UK; ^3^Stem Cell Group, Cardiovascular & Cell Sciences Research Institute, St. George's University of London, Cranmer Terrace, London SW17 0RE, UK; ^4^Department of Pharmacology, School of Pharmacy, University College London, 29-39 Brunswick Square, London WC1N 1AX, UK; ^5^Molecular and Cellular Immunology, UCL Institute of Child Health, 30 Guilford Street, London WC1N 1EH, UK; ^6^Antiviral Gene Therapy Research Unit, Faculty of Health Sciences, University of the Witwatersrand, Johannesburg, South Africa

## Abstract

Following fetal or neonatal gene transfer in mice and other species immune tolerance of the transgenic protein is frequently observed; however the underlying mechanisms remain largely undefined. In this study fetal and neonatal BALB/c mice received adenovirus vector to deliver human factor IX (hFIX) cDNA. The long-term tolerance of hFIX was robust in the face of immune challenge with hFIX protein and adjuvant but was eliminated by simultaneous administration of anti-CD25+ antibody. Naive irradiated BALB/c mice which had received lymphocytes from donors immunised with hFIX developed anti-hFIX antibodies upon immune challenge. Cotransplantation with CD4+CD25+ cells isolated from neonatally tolerized donors decreased the antibody response. In contrast, cotransplantation with CD4+CD25− cells isolated from the same donors increased the antibody response. These data provide evidence that immune tolerance following perinatal gene transfer is maintained by a CD4+CD25+ regulatory population.

## 1. Introduction

Fetal or neonatal gene transfer has been proposed as a potential therapeutic strategy with several advantages over adult gene transfer including increased likelihood of immune tolerization towards the transgenic protein [1, 2]. Absence of immune response after fetal or neonatal gene or stem cell delivery has been demonstrated in several studies [2–5], some of which have provided evidence for immune tolerance. We have previously observed prolonged expression of human factor IX (hFIX) after adenovirus-mediated gene transfer to fetal mice of the MF1 outbred strain. There was transient and minimal production of anti-hFIX antibodies after immune challenge by repeated injection of purified hFIX protein and hFIX-adenovirus in adulthood [6].

To interrogate the immune mechanisms after fetal or neonatal gene transfer more extensively we used inbred BALB/c mice. These mice were chosen since they express relatively high levels of hFIX after neonatal retroviral gene transfer [4], exhibit a dominant Th2 response [7], and, unlike outbred MF1 mice, can be used for adoptive transfer experiments. Adenovirus serotype 5 vector was used throughout.

## 2. Materials and Methods

Adenoviral vectors with CMV driving human factor FIX [6] (AdhFIX) and firefly luciferase [8] (AdLuc) genes were prepared. Vectors were titered by 293-cell plaque assays. E16 fetal mice received intravenous injection via the vitelline vessels [6]. P0 neonates received injections under hypothermic anaesthesia via the superficial temporal vein [9]. Adults received injections via the lateral tail vein. Fetuses, neonates, and adults received 1 × 10^9^, 1 × 10^9^, and 3 × 10^10^ vector genomes per mouse. Citrated blood was collected for plasma for hFIX assay and for serum for antibody analysis. Plasma concentrations of hFIX protein were determined using the Asserachrom FIX ELISA kit with pooled human plasma as assay standard. Antibodies against hFIX and adenovirus were detected as previously described [6]. Anti-CD25 IL2R rat IgG1 antibody was produced by PC61 hybridoma cells (American Type Culture Collection, Manassas, USA) using a capillary cell culture system as per manufacturer's protocol (CellMax Artificial Capillary Module, Cellco Inc., MD, USA). Antibody was purified and concentrated using affinity chromatography (Mab Trap, Amersham Biosciences UK Limited). For vector genome quantitation, 100 ng extracted DNA was subjected to quantitative PCR (ABI Prism 7900HT, Applied Biosystems Ltd., Warrington, UK) using hexon specific primers (5′CGCGGTGCGGCTGGTG3′ and 5′TGGCGCATCCCATTCTCC3′) normalized against a standard curve ranging from 10^1^ to 10^7^ adenovirus particles. For adoptive transfer experiments, recipients received sublethal irradiation (400 cGy) 24 h before cell transfer and hFIX immunization. Lymphocytes were harvested from inguinal, caudal, and mesenteric lymph nodes and combined with splenocytes. CD4+CD25+ and CD4+CD25− populations were purified using magnetic beads (MACS Cell Separation, Miltenyi Biotec, Surrey, UK). Purity was confirmed by FACS analysis. From sensitized or naive pooled donors 3 × 10^7^ lymphocytes were injected intravenously into recipients. 1 × 10^4^ or 1 × 10^5^ CD4+CD25+ or CD4+CD25− cells were cotransplanted; both concentrations were equally effective for the two transplanted populations; therefore antibody data was pooled for subsequent analysis and presentation. Luciferase expression in organ homogenates was assayed using the Luciferase Assay System (Promega, WI, USA) and luminometer (Lucy1, Anthos, Germany) as previously described [10].

## 3. Results

### 3.1. Longevity of Expression and Immune Challenge

**Figure 1:**
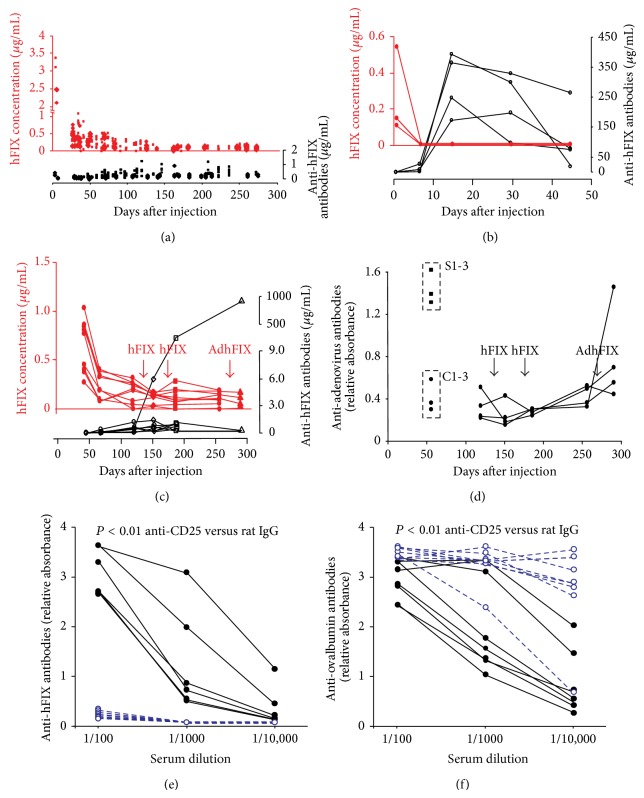
Measurement of concentrations of hFIX and anti-hFIX antibodies following perinatal and adult AdhFIX injection. (a) Concentrations of hFIX (red upper graph) and anti-hFIX antibodies (black lower graph) following fetal (*n* = 20, small squares) and neonatal (*n* = 21, large diamonds) injection of AdhFIX. (b) Concentrations of hFIX (red upper graph) and anti-hFIX antibodies (black lower graph) following adult injection of AdhFIX. (c) Concentrations of hFIX (red upper graph) and anti-hFIX antibodies (black lower graph) in mice injected* in utero* with AdhFIX. Two immune challenges with hFIX plus adjuvant and one AdhFIX were administered at *t* = 140, 175, and 275 (labelled arrows). (d) Concentration of anti-adenovirus antibody in naive controls (C1-3), positive control mice which received hFIX plus adjuvant (S1-3), and mice having received AdhFIX* in utero* (plotted lines). Mice having received AdhFIX* in utero* received immune challenges with hFIX plus adjuvant and one AdhFIX was administered at *t* = 140, 175, and 275 (labelled arrows). (e) Anti-hFIX antibodies in serially diluted plasma of mice tolerized to hFIX by fetal AdhFIX. Mice received three injections of anti-CD25+ depleting antibody (black, closed circles, and unbroken line) or rat IgG negative control (blue, open circles, and dotted line) simultaneously with hFIX plus ovalbumin plus adjuvant. Nine days after the final challenge, sera were collected for analysis. (f) Anti-ovalbumin antibodies in serially diluted plasma of mice tolerized to hFIX by fetal AdhFIX. Mice received three injections of anti-CD25+ depleting antibody (black, closed circles, and unbroken line) or rat IgG negative control (blue, open circles, and dotted line) simultaneously with hFIX plus ovalbumin plus adjuvant. Nine days after the final challenge, sera were collected for analysis.

We measured hFIX expression and anti-hFIX antibodies following intravascular injection of AdhFIX into fetal (*n* = 20), neonatal (*n* = 21) (Figure 1(a)), and adult (*n* = 4) (Figure 1(b)) BALB/c mice. After perinatal delivery hFIX concentrations were greater than 2 *μ*g/mL at 48 hours but declined to a stable concentration of approximately 50 ng/mL beyond 150 days; some mice (≈15%) lost expression. None developed significant anti-hFIX antibodies. In contrast, following adult delivery hFIX concentrations were between 0.1 and 0.6 *μ*g/mL at 48 hours and declined to zero within 7 days. High concentrations of anti-hFIX antibodies were detected from day 14 onwards.

As a stronger test of immune tolerance to hFIX, adult mice which had received AdhFIX* in utero* received two subcutaneous injections (*n* = 9) of purified hFIX plus MPL-TDM adjuvant (at 140 and 175 days of age) (four received an additional challenge of AdhFIX at 275 days (Figure 1(c)) to test tolerization to vector, described below). Eight mice showed no significant decrease in hFIX expression or increase in anti-hFIX antibodies after each challenge; however one mouse, which had no hFIX expression at the time of challenge, developed high titer anti-hFIX antibodies. This supports the observation of others that continual antigen expression is required for maintenance of tolerance following perinatal gene transfer [4]. All naive adult controls developed high anti-hFIX antibody titers following challenge (*n* = 4, data not shown).

### 3.2. Depletion of CD4+CD25+ Cells

The mechanism of immune tolerance was investigated. Six months after fetal AdhFIX injection, mice still expressing hFIX (range 24–148 ng/mL) received three intravenous injections of anti-CD25+ antibody (*n* = 7) to deplete CD25+ regulatory T cells; controls received rat IgG antibody (*n* = 7) (1 mg/mouse) (0, 4, and 29 days). Subsequent to each antibody injection, mice received a subcutaneous injection of a mix of hFIX (1 g/mouse), ovalbumin (1 g/mouse), and adjuvant (Freund's complete adjuvant and then 2x Freund's incomplete adjuvant). Blood was collected 9 days after the third immune challenge. All mice that received the anti-CD25+ antibody developed high anti-hFIX titres unlike those that received control rat IgG (Figure 1(e)). These results provide evidence that CD4+CD25+ regulatory T cells contribute to maintaining tolerance after perinatal gene transfer. To confirm that the depleting antibody was antigen-specific in breaking immune tolerance to hFIX rather than acting as a global immune stimulus, anti-ovalbumin antibody titres were measured. Unexpectedly, mice receiving depleting antibody developed a reduced immune response to ovalbumin (Figure 1(f)). Since PC61 also binds to recently activated cells, which express the IL-2 receptor *α*, those reactive to ovalbumin may be inactivated or deleted.

### 3.3. Adoptive Transfer Experiments

**Figure 2:**
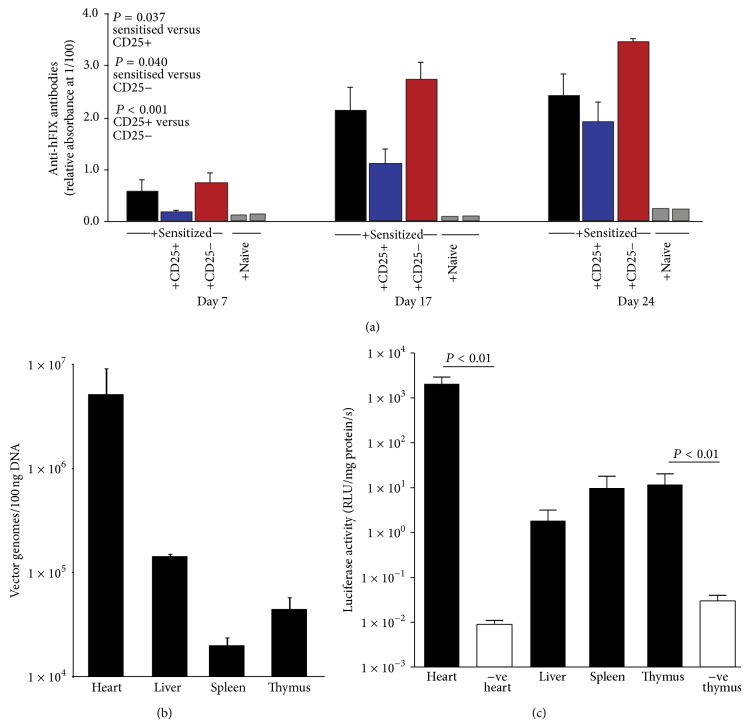
Adoptive transfer of regulatory cell populations, tissue distribution of vector, and transgene expression. (a) Anti-hFIX antibody concentrations in serum diluted 1/100 following adoptive transfer of lymphocytes from hFIX-sensitised mice (black bar), cotransplantation of sensitised cells plus CD4+CD25+ cells from neonatally tolerized mice (blue bar) or plus CD4+CD25− cells from neonatally tolerized mice (red bar), or adoptive transfer of lymphocytes from naive donors (grey bars). Recipients underwent sublethal irradiation, adoptive transfer, and hFIX-immunisation 24 hours later and blood collection on days 7, 17, and 24. (b) Quantitative PCR analysis of vector genome distribution and (c) luciferase expression six weeks after neonatal injection of AdLuc.

The mechanism of immune tolerance was interrogated further. A cohort of mice still expressing hFIX more than 250 days after neonatal AdhFIX injection received three subcutaneous immunisations with hFIX and Freund's adjuvant; immune tolerance was confirmed by hFIX expression and absence of antibodies. A second cohort of naive mice was sensitised by subcutaneous immunisation three times with hFIX and Freund's adjuvant. Irradiated BALB/c recipients received lymphocytes from sensitized mice (*n* = 4). Alternatively recipients received cotransplantation of sensitised lymphocytes plus either CD4+CD25+ (*n* = 9) or CD4+CD25− (*n* = 7) lymphocytes from tolerized mice (*n* = 9). Two recipients received whole lymphocyte population from naive donors. Following subcutaneous immunisation with hFIX and Freund's adjuvant blood was collected on days 7, 17, and 24 for anti-hFIX analysis (Figure 2(a)). At all times, recipients of sensitised cells produced substantially more anti-hFIX antibody than those which had received naive cells. Importantly, cotransplantation of CD4+CD25+ cells from tolerized mice significantly decreased antibody production (general linear model, Tukey pairwise comparison, *P* = 0.037) whereas cotransplantation of CD4+CD25− cells from tolerized mice significantly increased antibody production (*P* = 0.040). This provides further evidence that after perinatal gene transfer, a CD4+CD25+ population downregulates immune reactivity to hFIX as measured by anti-hFIX antibody production. This cell population appears to mediate tolerance in a greater number of gene therapy protocols than previously anticipated. For example, CD4+CD25+ regulatory T cells have been implicated in immune tolerance following liver gene transfer using adenoassociated virus vector [11]. Since cotransplantation of CD4+CD25− cells did not abolish antibody development in recipients, the role of other regulatory cell populations or deletional tolerance cannot be ruled out. Indeed, others provide evidence for deletional tolerance following injection of hFIX retrovirus into neonatal mice [12]. Nevertheless, recently, Gaensler and colleagues provided elegant demonstration for a role of CD4+CD25+ regulatory T cells in maintaining immune tolerance following neonatal intraperitoneal injection of adenoassociated virus vector [13].

### 3.4. Biodistribution of Vector Genomes and Gene Expression

Six to twelve weeks after neonatal AdLuc, luciferase expression and vector genomes were quantitated in different organs. Although fewer vector genomes were present in spleen (19,880 ± 4600 vg/100 ng DNA) and thymus (45,072 ± 14,693 vg/100 ng DNA) compared with those in other tissues (Figure 2(b)) significant luciferase expression was detected (Student's *t*-test, *P* < 0.01) (Figure 2(c)). Therefore, a role for central tolerance cannot be ruled out.

### 3.5. Evidence for Tolerization to Vector

Detection of anti-adenovirus antibodies was performed on four adult mice from the experiment shown in Figure 1(d) (i.e., they received AdhFIX* in utero*, two challenges of hFIX plus adjuvant on 140 and 175 days and AdhFIX on 275 days). Only one of these mice developed anti-adenovirus antibodies after adult AdhFIX challenge as did all the naive adult mice receiving AdhFIX challenge (S1-3) whereas antibodies remained undetectable in the remaining three, like in the nonchallenged controls (C1-3) (Figure 1(d)). This provides evidence for immune tolerization to vector components. This may be attributable to the use of first E1/E3-deleted adenovirus which might lead to continuous expression of small amounts of viral proteins, particularly since vector genomes can be detected in thymuses from adult mice injected neonatally with AdLuc (Figure 2(b)). Alternatively, it may arise from cotolerization from simultaneous initial exposure to hFIX and adenovirus antigens.

## 4. Discussion

Lifelong gene expression of various transgenes in mice has been achieved following perinatal delivery of vectors derived from adenovirus [6], gamma retrovirus [4], lentivirus [14], and adenoassociated virus [15, 16]. These studies have led to improvements in the vector and expression cassette and provide motivation for childhood or even infant gene therapy for early onset genetic diseases. However understanding of the immune response to perinatal gene therapy remains poor. Here, we provide evidence for a contribution of regulatory tolerance to transgenic proteins following fetal and neonatal delivery in BALB/c mice. This supports recent observations of regulatory tolerance following AAV vector delivery [13]. One unexpected finding was evidence for immune tolerance to vector components. Although this might permit multiple postnatal administrations of the same vector, it might also render the recipient vulnerable to infection by the archetypal virus, clearly an undesirable outcome. One possible explanation for tolerization is residual and continuous expression of viral proteins. This provides strong justification for removal of all viral sequences from vectors designed for long-term treatment of genetic diseases. For example, third generation HIV-based vectors contain approximately 1.8 kb of viral genetic material. This situation contrasts with recent observations by Carlon and colleagues, where perinatal delivery of AAV vector to murine airways benefitted from immune ignorance (rather than tolerance) to the capsid proteins, permitting a second administration at a later time point [17].

In these studies, it is important to consider the developmental stage of the mouse immune system in late gestation relative to the immune system of the fetal and newborn human. Holladay and Smialowicz have provided an excellent comparison of the developing mouse and human immune system. By the end of the third trimester, the human B cells express surface IgG, lymphocyte precursors are present in the thymus, thymocytes subsequently become responsive to mitogens, and functional natural killer cells arise. In contrast, in the mouse, lymphocyte precursors in the thymus are detected at around 11 days of gestation whereas B cell surface IgG and thymocytes responsiveness to mitogen occurs around 17 days of gestation and NK cells arise after birth [18]. In humans, mature peripheral a/b T cells first appear towards the end of the first trimester whereas in mice they only appear at the end of gestation [19]. However the situation is more complex, in that human serum IgM and IgG concentrations do not reach adult concentrations until 1-2 years of age and 4–6 years of age, respectively. Moreover before 2 years of age, babies fail to mount humoral immune responses to some antigens [18]. An additional complication is that, recently, it has been shown that in humans, as in mice and birds, the immune system is layered, whereby hematopoiesis occurs in waves, specifically an early one which gives rise to fetal T cells and a later one that gives rise to different adult T cells. The fetal and adult T cells possess different functional properties and gene expression patterns [20].

BALB/c mice produce relatively small litters and frequently cannibalise their litters following maternal surgery. For reasons of cost and to minimise animal wastage we switched from fetal to neonatal gene transfer for adoptive T cell transfer experiments as neonatal death following gene transfer is very rare. Nevertheless it is important to recognise that both qualitative and quantitative differences in immune function between E16 fetal and P0 newborn mouse exist.

Over the course of the study we switched from MPL-TDM adjuvant to Freund's adjuvant. MPL-TDM is considered to pose less of a risk of abscess formation and is therefore a better choice when complying with the 3Rs principle of refining experiments to minimise animal pain and distress. However we chose to use Freund's adjuvant for experiments involving regulatory T cell depletion and adoptive T cell transfer, as Freund's adjuvant was considered likely to provide the most potent challenge [21].

*Ex vivo* luciferase expression correlated poorly with vector copy number. This may be explained by the observation that hemoglobin interferes substantially with* ex vivo* luminometry. This is particularly apparent in the spleen which retains a particularly high concentration of red blood cells and hemoglobin even when perfused [22]. An additional explanation may be that the CMV promoter which drives luciferase expression in this vector may vary in activity in different tissue types.
